# Molecular genetics and phenotypic assessment of foxtail millet (*Setaria italica* (L.) P. Beauv.) landraces revealed remarkable variability of morpho-physiological, yield, and yield‐related traits

**DOI:** 10.3389/fgene.2023.1052575

**Published:** 2023-01-25

**Authors:** Palakurthi Ramesh, Vijaya Naresh Juturu, Poli Yugandhar, Sydney Pedersen, Alavilli Hemasundar, Seher Yolcu, Puli Chandra Obul Reddy, C. V. Chandra Mohan Reddy, P. Veerabramha Chari, Rajinikanth Mohan, Akila Chandra Sekhar

**Affiliations:** ^1^ Molecular Genetics and Functional Genomics Laboratory, Department of Biotechnology, School of Life Sciences, Yogi Vemana University, Kadapa, Andhra Pradesh, India; ^2^ Plant Molecular Biology Laboratory, Indian Institute of Rice Research, Hyderabad, Telangana, India; ^3^ Department of Biology, Mercyhurst University, Erie, PA, United States; ^4^ Department of Bioresources Engineering, Sejong University, Seoul, South Korea; ^5^ Department of Life Sciences, Sogang University, Seoul, South Korea; ^6^ Plant Molecular Biology Laboratory, Department of Botany, School of Life Sciences, Yogi Vemana University, Kadapa, Andhra Pradesh, India; ^7^ ANGRAU—Agriculture Research Station, Anantapur, Andhra Pradesh, India; ^8^ Department of Biotechnology, Krishna University, Machilipatnam, Andhra Pradesh, India

**Keywords:** foxtail millet, landrace, multivariate statistical analysis, yield, yield associated traits, genetic diversity, SSR markers, crop improvement

## Abstract

Foxtail millet (*Setaria italica* (L.) P. Beauv.) is highly valued for nutritional traits, stress tolerance and sustainability in resource-poor dryland agriculture. However, the low productivity of this crop in semi-arid regions of Southern India, is further threatened by climate stress. Landraces are valuable genetic resources, regionally adapted in form of novel alleles that are responsible for cope up the adverse conditions used by local farmers. In recent years, there is an erosion of genetic diversity. We have hypothesized that plant genetic resources collected from the semi-arid climatic zone would serve as a source of novel alleles for the development of climate resilience foxtail millet lines with enhanced yield. Keeping in view, there is an urgent need for conservation of genetic resources. To explore the genetic diversity, to identify superior genotypes and novel alleles, we collected a heterogeneous mixture of foxtail millet landraces from farmer fields. In an extensive multi-year study, we developed twenty genetically fixed foxtail millet landraces by single seed descent method. These landraces characterized along with four released cultivars with agro-morphological, physiological, yield and yield-related traits assessed genetic diversity and population structure. The landraces showed significant diversity in all the studied traits. We identified landraces S3G5, Red, Black and S1C1 that showed outstanding grain yield with earlier flowering, and maturity as compared to released cultivars. Diversity analysis using 67 simple sequence repeat microsatellite and other markers detected 127 alleles including 11 rare alleles, averaging 1.89 alleles per locus, expected heterozygosity of 0.26 and an average polymorphism information content of 0.23, collectively indicating a moderate genetic diversity in the landrace populations. Euclidean Ward’s clustering, based on the molecular markers, principal coordinate analysis and structure analysis concordantly distinguished the genotypes into two to three sub-populations. A significant phenotypic and genotypic diversity observed in the landraces indicates a diverse gene pool that can be utilized for sustainable foxtail millet crop improvement.

## Introduction

Millets are among the oldest cultivated cereals that carry great importance in agriculture. Among the minor millets, foxtail millet is an ancient diploid, C4 cereal crop from Eurasia (Sakamoto, 1987; Yang et al., 2020). It is a staple crop in many parts of the world and used for daily caloric intake, feed, and fodder in many parts of Africa and Asia. For a cereal, foxtail millet grains have a relatively high protein content; in addition, their gluten-free nature and low glycemic index makes them desirable (Goudar et al., 2011; Yang et al., 2022). Foxtail millet ranks second in millet production with an average of six million tons from the Southern part of Europe and Asia alone (Yang et al., 2012; Aidoo et al., 2016). Globally it is cultivated in marginal and resource poor soils and is an ideal crop for sustainable agriculture (Meng et al., 2020). The short life cycle, high breeding rate and minimal water for rapid maturation (Doust et al., 2009; Xing et al., 2022) make foxtail millet a farmer’s favorite. In addition, the hardiness of this plant makes it an excellent model crop for genetic and molecular research on water use efficiency and drought tolerance, especially in the context of C4 metabolism (Doust et al., 2009; Li and Brutnell, 2011). Foxtail millet genome was sequenced by two independent groups (Bennetzen et al., 2012; Zhang et al., 2012).

Landraces are locally adapted domesticated species often used by local farmers, serving as valuable genetic resources for sustainable agriculture. Landraces are generally known to yield less compared to released cultivars but may carry superior stress tolerance (Mohammadi et al., 2015; Dwivedi et al., 2016; Shivhare and Lata, 2017; Azeez et al., 2018; Pradhan et al., 2020). The low yield of foxtail millet, compared to other cereal crops, is associated with various agronomic, physiological, and genetic factors (Nirmalakumari and Vetriventhan, 2010). Improvement of landraces for higher yield as well as better adaptability to various environmental stresses can augment crop production and farmer incomes. In addition, tagging the genetic diversity of the germplasm within local and regional collections using molecular markers can aid in discovery of novel genes for various phenotypic and stress-related traits (Hegde and Mishra, 2009).

Even though 26,670 accessions of foxtail millet from 26 countries are stored at the Chinese National Gene Bank (CNGB), they are not easily accessible outside of China (Wang et al., 2012) and 1,474 foxtail millet germplasm from 23 countries are maintained in the International Crops Research Institute for the Semi-Arid Tropics (ICRISAT), gene bank (Upadhyaya et al., 2009).

In comparison, in India, far fewer landraces of foxtail millet are documented (∼1,500), yet this crop is extensively grown in semiarid states of Andhra Pradesh, Karnataka, Tamil Nadu, Uttar Pradesh, Uttarakhand, Maharashtra, Rajasthan, Gujarat and North Eastern States (Kumari et al., 2011). Therefore, new germplasm collections are imperative to document the genetic diversity of foxtail millet in India (Li et al., 1998; Goron and Raizada, 2015). Isozyme and DNA based studies among the millets revealed a higher genetic diversity in green millet (*S. viridis*) and foxtail millet (Wang et al., 1995; Le Thierry d’Ennequin et al., 2000). Estimation of diversity in landraces using molecular markers was initiated long ago in foxtail millet (Fukunaga et al., 2002; Hirano et al., 2011; Kim et al., 2012; Wang et al., 2012; Bai et al., 2013; Ghimire et al., 2018; Liu et al., 2019), but studies in India are scant (Kumari et al., 2011). Simple sequence repeat (SSR) marker are excellent tools to study genetic diversity and mapping of associated genes for various qualitative and quantitative traits (Jia et al., 2009; Sato et al., 2013; Goron and Raizada, 2015; Ramakrishnan et al., 2016; Zhu et al., 2016; Moharil et al., 2019). Compared to other markers, SSR markers are more suitable because of their extensive genome coverage, high polymorphism, and reproducibility (Soriano et al., 2016). Previous reports demonstrated the use of SSR markers for genetic diversity studies in foxtail millet (Jia et al., 2009; Liu et al., 2011; Kim et al., 2012; Vetriventhan et al., 2012). Functional markers like Expressed Sequence Tag (EST)-SSRs and Transposable Elements (TEs) based markers have also been extensively used to study genetic variability (Wang et al., 2012; Pandey et al., 2013; Gupta et al., 2014).

In recent years, there is a scientific and social concern on the erosion of genetic diversity in biological systems, in specific, the crop diversity of genes, species and their ecosystems. Agro-biodiversity plays a major role to deal with this challenge. Keeping in view, there is an urgent need for conservation of genetic resources. To our knowledge, less efforts have been made for the systematic collection, agro-morphological, physiological, yield and yield-related traits and molecular markers characterization of foxtail millet landraces from local farmer fields who are not yet adapted the modern released/improved cultivars. With this background we hypothesized the necessity of the systematic characterization of the land races from a semi-arid tropic region would provide a novel genetic source for improvement of foxtail millet. These landraces will have novel allelic variants to cope up with extreme environmental conditions with enhanced yield. The objectives of the study is to develop genetically fixed foxtail millet landraces from a heterogeneous mixture and assess the genetic diversity based on agro-morphological, physiological, yield and yield-related traits and molecular markers. Hence, the present study has been carried to systematically characterize gathered heterogeneous mixture of foxtail millet landraces from local farmer fields in semi-arid region of Rayalaseema in South India. The collections were separated using the Single Seed Descent (SSD) method for eight generations, after which the lines were characterized based on morpho-physiological, yield and yield related traits and molecular markers along with four released cultivars. In the process, we identified superior high yielding landraces and genetic diversity that could be tapped for future breeding and research.

## Materials and methods

### Plant materials

The foxtail millet landraces were grown in a greenhouse at a day/night temperature of 30°C ± 1°C/37°C ± 1°C and relative humidity varied from 50%–80% at Yogi Vemana University, Kadapa, Andhra Pradesh (latitude of 14°.47′N and longitude 78°.71′E at a sea level of 90 m). Each genotype was grown in three rows with two replications with intra and inter-row spacing of 5 and 22.5 cm, respectively, and a depth of 2–3 cm (https://milletadvisor.com/foxtail-millet-farming/). In each genotype, 10 individuals with uniform traits were tagged and harvested separately and evaluated for the morpho-physiological, yield and yield related traits. For simplicity, the four major locations-Ipperu, Korrapadu, Chittoor and Madikera are designated as location 1 (S1), location 2 (S2), location 3 (S3) and location 4 (S4), respectively. The G in the genotype designations represents Green and the C indicates Color; the latter represents plants that display coloration in at least one of the following regions: culm, stem base, leaf sheath, panicle or bristles. Based on seed coat color, the genotype from Basavanapalli is designated as Black and the one from Punganur is designated as Red (Supplementary Table S1).

### Development of landraces

Mixed lines of indigenous foxtail millet germplasm were collected from farmers’ fields in the semi-arid region of Rayalaseema, Andhra Pradesh in South India (Supplementary Table S1). In subsequent generations these heterogeneous mixtures of foxtail millet landraces were evaluated in a greenhouse and separated based on morphological trait variations and named primarily according to their color traits for each subsequent generations up to eight generations, the lines were perpetuated using the SSD method and trait variations were noted. The segregation of these color traits in all generations was based on blind selection of a single panicle out of many others individuals, this was done for eight generations. To serve as a reference for the landraces we developed, four released varieties were obtained from Regional Agricultural Research Station, Nandyal, India and included in this study.

### Phenotyping

A total of 28 morpho-physiological, yield and yield-related traits including 9 qualitative and 19 quantitative traits such as stem base color (SBC), leaf size (LS), panicle lobbing (PB), inflorescence compactness (IC), bristles (BSL), type of bristles (TB), color of bristles (CB), apex sterility (APX), seed color (SC), root angle @1 (RA1, cm), root angle @2 (RA2, cm), root angle @3 (RA3, cm), root angle @4 (RA4, cm), plant height (PHT, cm), plant dry weight (PDW, g/plant), days to 50% flowering (D50%F), days to maturity (DM), panicle exertion (PE, cm), panicle length (PL, cm), panicle weight (PWT, g), total seed weight (TSW, g), 1000 seed weight (1000SDW, g), chlorophyll content (SPAD cc), number of stomata (NS), number of epidermal cells (NE), stomatal index (SI), relative water content (RWC), ion leakage (ILN) were used in this study and descriptor traits of foxtail millet for characterization given in Table 1. The data were recorded from 10 tagged individual lines in each replication. For counting two of the physiological traits, the NS and NEC clear nail polish was applied on the abaxial and adaxial leaf surfaces. After drying, the nail polish layer was peeled carefully from the leaf surface leaving a leaf impression and transferred onto a glass slide. The leaf impression was then covered and observed under a microscope. NS and NEC were counted under magnification of ×10, an average of three such readings at different regions from the leaf impression were considered. Qualitative traits were coded as binary and non-binary descriptions for principal components analysis (PCA) and two-way cluster analysis, respectively. Colored bars indicated in the two-way cluster corresponding to the trait mean data.

**TABLE 1:** List of the 28 morpho-physiological, yield and yield related traits used in the study.

S. No.	Qualitative trait name	Abbreviation	Trait description
1	Stem base color	SBC	Pink and green
2	Leaf size	LS	Small, medium and broad
3	Panicle lobing	PB	Non-lobed and medium lobed
4	Inflorescence compactness	IC	Compact, medium and loose
5	Bristles	BSL	Present and absent
6	Type of bristles	TB	Small, medium and long
7	Color of bristles	CB	Pink and green
8	Apexsterility	AST	Present and absent
9	Seed color	SC	Yellow, red and black
	Quantitative trait name	Abbreviation	Trait description
1	Root angle @1 (cm)	RA1	Recorded at the harvest stage, measured the point from base of stem to below 1 cm level
2	Root angle @2 (cm)	RA2	Recorded at the harvest stage, measured the point from base of stem to below 2 cm level
3	Root angle @3 (cm)	RA3	Recorded at the harvest stage, measured the point from base of stem to below 3 cm level
4	Root angle @4 (cm)	RA4	Recorded at the harvest stage, measured the point from base of stem to below 4 cm level
5	Plant height (cm)	PHT	Recorded at 50% flowering stage, measured as the distance from ground level to the main panicle/flag leaf tip
6	Plant dry weight (g/plant)	PDW	Recorded at the harvest stage, plant materials were dried at 80 ᵒC for 3 days, and then dry weight was recorded
7	Days to 50% flowering	DF	Number of days from sowing to approximately 50% of plants were at the flowering stage
8	Days to maturity	DM	Physiological maturity was recorded when 80% of the plants were dried/matured
9	Panicle exertion (cm)	PE	Recorded at the maturity stage, measured from the point flag leaf to the base of the main panicle
10	Panicle length (cm)	PL	Recorded at the maturity stage, length of the panicle base to the tip of panicle excluding bristles
11	Panicle weight (g)	PWT	Recorded after harvesting by measuring the total weight of the panicle
12	Total seed weight (g)	TSDW	Recorded after harvesting by crushing the panicle and measured as total grains obtained
13	1,000 seed weight (g)	1000SDW	Recorded after harvesting by crushing the panicle and 1,000 randomly selected grains
	Physiology traits		
14	Soil Plant Analysis Development chlorophyll content	SPADcc	Recorded during 9.00–11.00 a.m. as described by Nageswara Rao et al. (2001)
15	Number of stomata	NS	Counted at 25 days after seed sowing
16	Number of epidermal cells	NEC	Counted at 25 days after seed sowing
17	Stomatal index	SI	Calculated according to the method described by Salisbury, (1927)
18	Relative water content	RWC	Observed at 25 days after seed sowing as described in Gonzalez and Gonzanlez-Vilar (2001)
19	Ion leakage	ILG	Carried out by using third, fully expanded leaf at 25 days after seed sowing, as described in Bajji et al. (2002)

### DNA isolation and genotyping

DNA was isolated from leaves using the CTAB method described by Murray and Thompson (1980). A total of 34 SSR, 21 EST SSR, 9 TE based, and 3 rice SSR markers were used in this study (Supplementary Table S2). The PCR reaction was carried out in Thermal Cycler (Eppendorf master cycler pro AG 6321, Germany) with reaction mixture of 20 μL containing approximately 40 ng template genomic DNA, 1×PCR buffer (10 mM Tris-HCl, pH 8.3, 500 mM KCl), 1.5 mM MgCl2, 10 pmol of each primer, 0.12 mM of each dNTP, and 0.3 U of Taq DNA polymerase. PCR was carried out by the following protocol: 2 min and 30 s at 95°C (initial denaturation step), followed by 35 cycles at 94°C for 60 s, 49°C–55°C for 60 s, 72°C for 1 min, and a final extension at 72°C for 8 min. The resulting amplification products were resolved on 3% agarose gels prepared with 1X TAE buffer and electrophoresis was carried out at 110 V for 1 h. Ethidium bromide-stained gels were observed under UV gel documentation system. Clear and explicit bands were used for scoring. The bands were scored for the presence (1) or absence (0) for the corresponding genotypes and formed data matrix with 1 or 0 for genotyping.

### Statistical analysis

All the statistical analysis among and between the variants was determined by Analysis of Variance (ANOVA) by using Indostat 9.1 software. The same software was used for broad-sense heritability (H2), genetic advance and Path coefficient analysis. Correlation analysis was performed using R (R, Development Core Team, 2012) and PCA and two-way cluster analysis were done using SAS JMP statistical discovery software (SAS, 2012). Genetic relatedness among and between the genotypes were determined by using Popgene version 1.31 (Yeh et al., 1999). Polymorphism Information Content (PIC) value and Principal Coordinate Analysis (PCoA) was determined by using GenAlEx package (Peakall and Smouse, 2012). Diversity within the germplasm/selected lines was calculated by Euclidean Ward’s method by using SAS JMP statistical discovery software (SAS, 2012). STRUCTURE 2.3.4 program was implemented with burn period of 10,000 and run length 100,000 Markov Chain Monte Carlo number (MCMC) for analysis of population structure with K = 3 to 10. Best K value was identified by using Structure Harvester (Earl and VonHoldt, 2012) and Ln probability was used for identification of genetic diversity in the population using graphical approach (Evanno et al., 2005).

## Results

### Development of landraces

**FIGURE 1:**
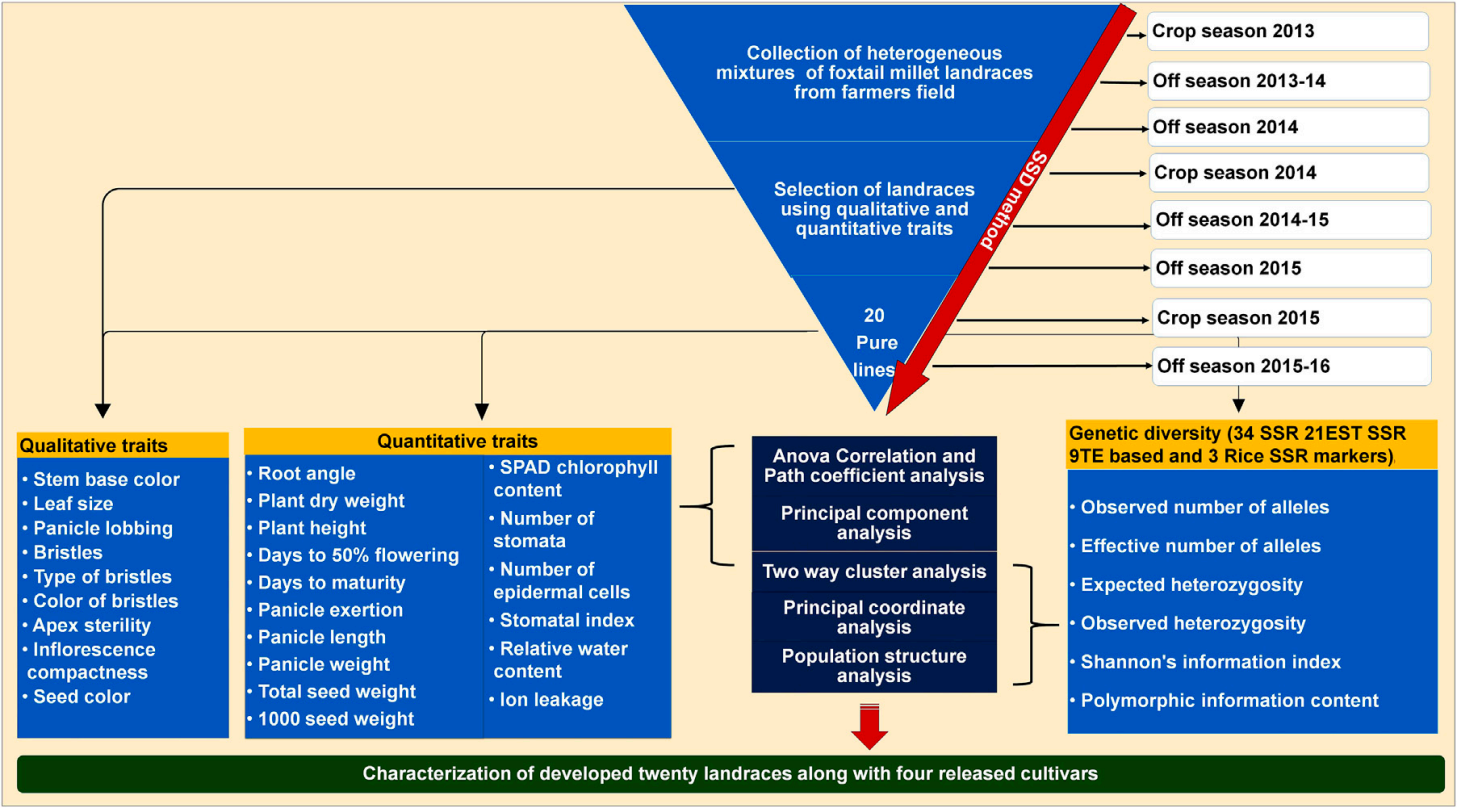
Scheme for twenty foxtail millet landraces development process based on SSD method using from collected heterogeneous mixture in farmer fields. Details of the characterization of these landraces along with released cultivars based on qualitative, quantitative traits and molecular markers.

In order to develop genetically fixed landraces, heterogeneous mixture of foxtail millet landraces were collected from farmer fields and subjected to SSD method for eight generations to develop pure lines with stabilized traits. Four commercial varieties/released cultivars (Krishnadevaraya, Narashimharaya, Prasad and Srilaxmi) were used for trait comparison. A detailed scheme of the landrace development and characterization with released cultivars presented in Figure 1. Significant variability was noticed within the developed lines and large (morpho-physiological, yield and yield related traits) variation among the developed landraces.

### Phenotypic performance

**FIGURE 2:**
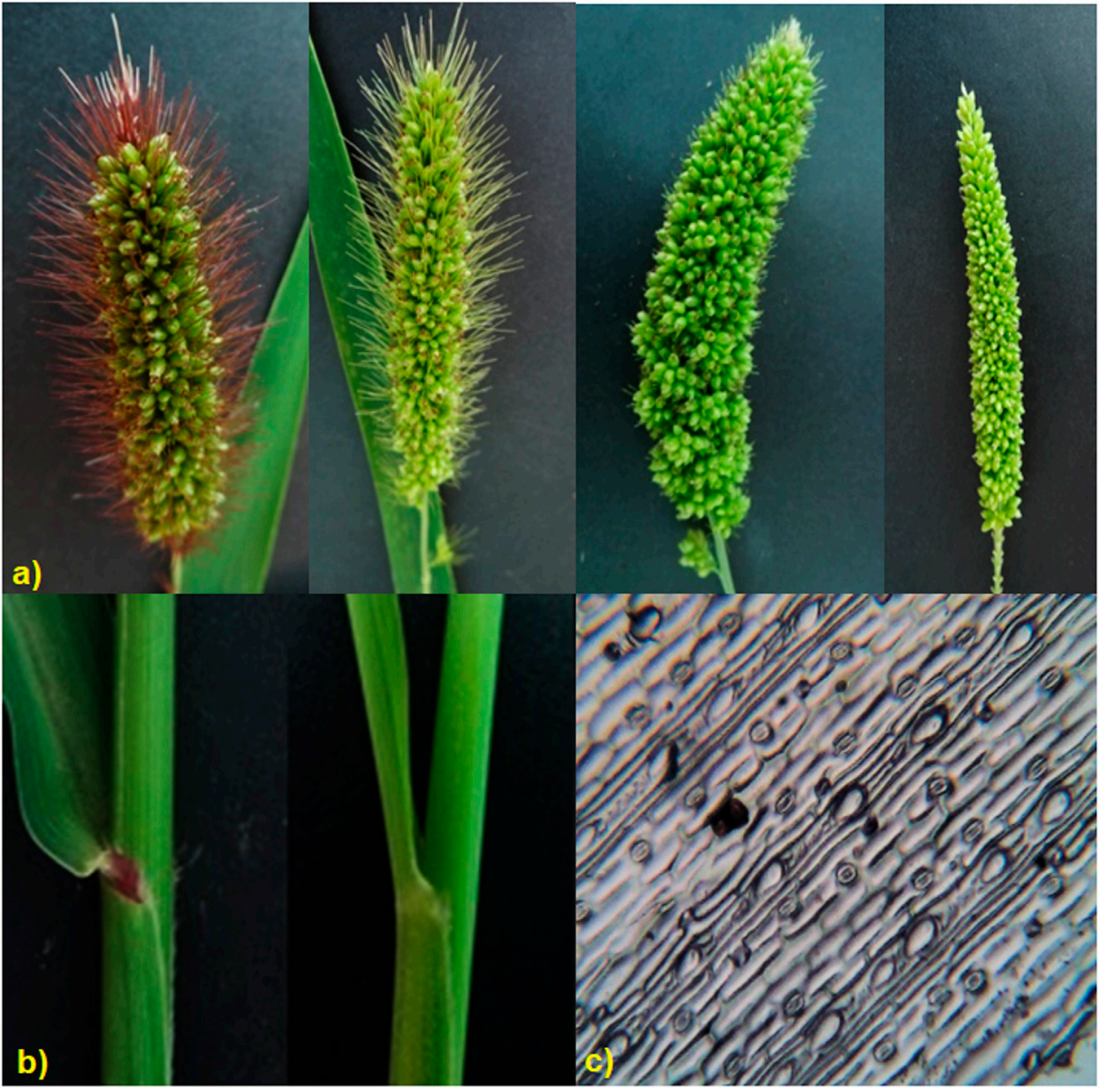
Morphological, yield and yield related traits measured in twenty foxtail millet landraces along with four released cultivars **(A)** panicle variation, **(B)** leaf sheath color variation, **(C)** Stomatal and epidermal cell variation.

Remarkable phenotypic variation was observed in 28 studied traits including 9 qualitative and 19 quantitative traits among the landraces along with four released cultivars (Figure 2; Supplementary Figures S2, S3; Supplementary Tables S3, S4). Significant variation was found for the traits PHT (61–102 cm) and D50%F (42–54), DM (64–77), PE (4.22–19 cm), PL (4.5–9.72 cm), PWT (0.86–1.84 g) and TSDW (0.73–1.46 g). ANOVA for the 19 quantitative traits indicated a significant difference among the 20 foxtail millet landraces along with four released cultivars at *p* < 0.05 and *p* < 0.01 (Table 2). The highest phenotypic variation was observed for PWT, PE, PLT and PDW. The estimated highest genotypic coefficient of variation (GCV) with high phenotypic coefficient of variation (PCV) in PWT followed by PE, PDW, RA1, RA2, RA3, RA4, and TSDW respectively. The estimated H2 value coupled with high genetic advance were observed for DF, DM, SI and TSDW. This clearly indicates that traits were affected by genetic factors (Table 3).

**TABLE 2:** Analysis of variance of studied 19 quantitative traits were evaluated on 20 foxtail millet landraces along with four released cultivars.

	PHT	PDW	DF	DM	PE	PL	PWT	TSDW	1000SDW
Minimum	61.590	1.455	42.500	64.500	4.220	4.560	0.865	0.730	2.060
Maximum	102.350	3.345	54.000	77.500	19.000	9.720	1.840	1.460	3.220
Mean	80.588	2.322	47.792	70.750	11.012	6.809	1.219	1.222	2.930
SS	7,086.610	19.443	475.917	633.000	487.180	95.421	4.775	2.114	2.860
MS	308.113	0.845	20.692	27.522	21.182	4.149	0.208	0.092	0.124
F	9,182.020	40.390			1,044.860	267.520	13.370	9.460	19.140
*p*	0.001	0.001	0.001	0.001	0.001	0.001	0.001	0.001	0.001
CV	0.230	6.230			1.290	1.830	10.220	8.070	2.750

	RA1	RA2	RA3	RA4	ION	SCC	SI	NEC	NS	RWC
Minimum	1.940	1.650	1.800	1.890	1.145	35.925	17.640	217.000	58.000	70.250
Maximum	3.300	3.700	4.350	4.300	5.060	48.900	34.345	428.000	107.000	91.435
Mean	2.673	2.923	3.172	3.119	2.529	41.107	24.265	297.630	71.542	81.389
SS	6.000	11.459	18.398	19.371	51.867	499.304	784.529	120,363.000	5,175.920	1,275.340
MS	0.261	0.498	0.800	0.842	2.255	21.709	34.110	5,233.180	225.040	55.450
F	20.790	4.840	114.560	64.840	267.540	1,381.270	400.490	4,150.460	163.450	4,165.070
P	0.001	0.001	0.001	0.001	0.001	0.001	0.001	0.001	0.001	0.001
CV	4.190	10.980	2.630	3.650	3.630	0.300	1.200	0.380	1.640	0.140

Note: Significant at 0.05 probability level. Significant at 0.01 probability level.

SS, sum of squares; MS, mean of squares.

**TABLE 3:** Genetic variability parameters for 19 quantitative traits evaluated on 20 foxtail millet landraces along with four released cultivars.

Trait	h^2^ (broad sense)	Gen. Adv 5%	Gen. Adv 1%	Gen. Adv as % of mean 5%	Gen. Adv as % of mean 1%	ECV	GCV	PCV
Plant height	32	7.4	9.49	9.91	12.7	12.1	8.42	14.8
Plant dry weight	100	1.34	1.72	57.9	74.2	0	28.1	28.1
Days to flower	100	6.62	8.49	13.9	17.8	0	6.73	6.73
Days to Maturity	100	7.64	9.79	10.8	13.8	0	5.24	5.24
Panicle Exertion	21	1.24	1.6	14.2	18.3	28.9	15	32.6
Panicle Length	44	2.17	2.78	26.9	34.4	22.1	19.6	29.5
Panicle weight	42	0.41	0.52	28.8	36.9	25.1	21.5	33
1,000 seed weight	98	0.48	0.61	16.4	21.1	1.03	8.04	8.1
Total seed weight	99	0.42	0.54	34.4	44.1	0.49	16.7	16.7
Root angle 1	100	0.74	0.95	27.8	35.6	0	13.5	13.5
Root angle 2	100	1.01	1.3	33.9	43.5	0	16.5	16.5
Root angle 3	100	1.3	1.67	41.1	52.7	0	20	20
Root angle 4	100	1.31	1.69	42.4	54.4	0	20.6	20.6
SPAB chlorophyll Content	84	6.38	8.18	15.6	19.9	3.53	8.21	8.94
No of stomata	70	17.1	21.9	23.8	30.6	8.8	13.7	16.3
No of epidermal cell	77	77.1	98.8	25.9	33.2	7.66	14.3	16.2
Stomatal index	100	8.53	8.53	35	44.9	0	17	17
RWC	100	10.8	10.8	13.3	17.1	0	6.46	6.46
Ion leakage	100	1.19	1.19	9.34	14.9	0	7.82	7.82

Gen. Adv 5%, Genetic Advancement at 5% level; Gen. Adv 1%, Genetic Advancement at 1% level; Gen. Adv as % of Mean 5%, Genetic Advancement as percentage of mean 5% level; Gen. Adv as % of Mean 1%, Genetic Advancement as percentage of mean 1% level; ECV, environmental coefficient of variation; GCV, genotypic coefficient of variation PCV, phenotypic coefficient of variation.

### Correlation and path coefficient analysis

**FIGURE 3:**
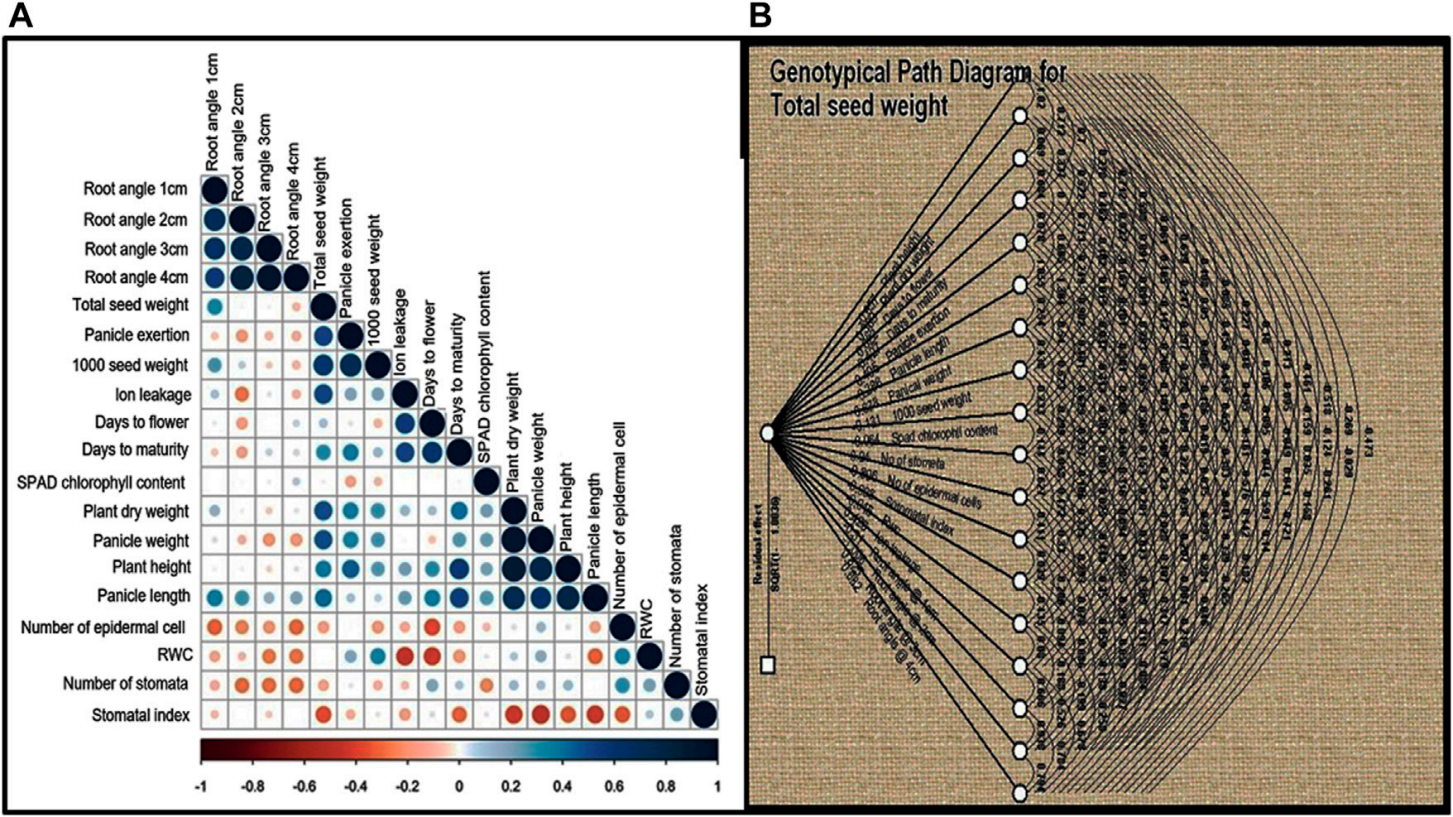
**(A)** Correlation analysis was performed for 19 quantitative traits for twenty foxtail millet landraces along with four released cultivars. Dark blue circles represents significant positive correlation, brown circles represents significant negative correlation and empty circles represent no significant correlation. Correlation with *p* < 0.05 was taken as statistically significant. **(B)** Path coefficient analysis of direct and indirect effects of 19 quantitative traits (path coefficient) on total seed yield. Double and single-arrowed lines represents mutual and direct influence among the morphological traits.

In order to understand the nature and degree of relationships between grain yield and other quantitative traits, Pearson’s correlation coefficients (r) were calculated for the 19 quantitative traits and were found to be statistically significant (*p* < 0.001), as shown in Figure 3A TSDW showed significant positive associations at *p* < 0.05 to 0.001 with traits like RA1, RA2, RA3, but not RA4, PHT, PDW, D50%F, DM and panicle characteristics like PE, PL and PWT. TSDW also showed significant negative associations with the almost all physiological traits i.e., SPADcc, NS, NEC SI and RWC (except ILG) (Figure 3A). Since correlation analysis does not explain potential cause and effect, path coefficient analysis was performed with total seed weight/seed yield as a dependent variable. In Figure 3B, double and single-arrowed lines represent mutual and direct influence among the morphological and physiological traits as measured by path coefficients that are presented in Supplementary Table S5. Number of stomata contributed highest positive direct effect to TSDW (0.93) followed by PDW (0.38), D50%F (0.62), PE (0.40), PWT (0.67), ILG (0.42), RA 3 (0.82) and RA 4 (0.68). Further, RA 2 contributed the highest negative direct effect to TSDW (−1.00). SPAD cc (−0.06), RWC (−0.01), RA1 (−0.30), SI (−0.99), NEC (−0.80), 1000SDW (−0.13), PL (−0.28) and PHT (−0.65). Most of the traits showed an indirect positive or negative effect with TSDW (Figure 3B; Supplementary Table S5). Since both correlation and path coefficient analysis demonstrated that D50%F and DM correlated strongly and directly affected grain yield, we identified four relatively early flowering and maturing, high yielding landraces that had yield on par with or surpassing the released cultivars- S1C1, S3G5, Red and Black (Supplementary Tables S4, S6). Based on both analyses, RA 3, PDW, D50%F, DM, PWT and PE correlate with and directly impact seed yield and therefore, may be used as factors in selecting high yielding genotypes of foxtail millet.

### Principal components analysis (PCA) for qualitative and quantitative traits

**FIGURE 4:**
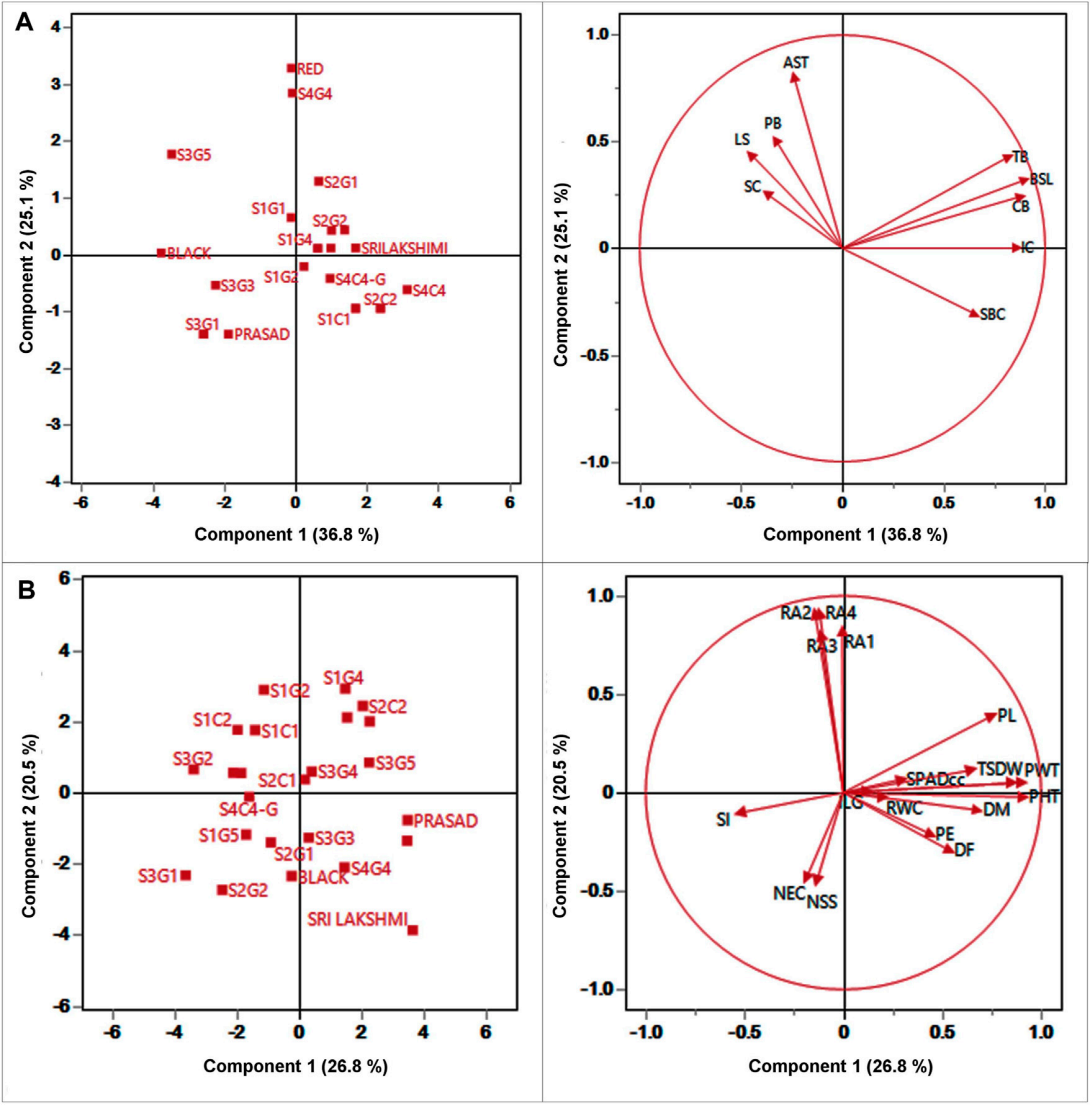
Plot for the first (PC1), and second (PC2) of the principal component analysis displaying the distribution of twenty foxtail millet landraces along with four released cultivars and **(A)** 9 qualitative traits **(B)** 19 quantitative traits.

PCA analysis was performed to identify the sources of variation among the different landraces. PCA reveals the similarities and differences among the genotypes based on contribution of each qualitative and quantitative trait to the total variance. PCA can also aid in the identification of traits that contribute significantly in partitioning the 24 foxtail millet genotypes based on the 28 studied traits. A total of nine qualitative traits were subjected to PCA with four principal components (eigenvalues ≥1) accounting for 84.93% of the variation (Figure 4A). The first three components of PCA largely encompassed the traits that distinguished the landraces. PC 1 was contributed by SBC, BSL, TB, and CB which accounted for 36.8% of total variation. LS and SC were the most important contributors to PC 2, which accounted for 25.07% of the total variation. The major characters that contributed to PC three were APX and PB which accounted for 15.21% of the total variation (Figure 4A; Supplementary Table S7). As the biplot reveals, the bristles traits-presence or absence of BSL, TB and CB strongly correlated with each other and also correlated positively with IC and to a lesser extent with SBC. All these traits correlated negatively with the remaining four traits AST, PB, LS and SC which associated positively with each other.

Likewise, 19 quantitative traits were also used to perform PCA; PC 1 was contributed by PHT, PDW, D50%F, DM, PE, PL, PWT, TSDW, SPADcc and ILG (Figure 4B; Supplementary Table S8) which accounted for 26.75% of the total genetic variance. Root angles (RA1, RA2, RA3 and RA4) were the most important contributors to PC 2 with 20.52% of the total variation. The major contributors to PC three were the physiological traits, NS, NEC and RWC, with 12.61% of the total variation. The PCA also reveals that TSDW had a strong correlation with PHT, PDW, DM, PWT, and SPADcc. Furthermore, TSDW also correlated positively with PL, PE and D50%F, consistent with correlation and path coefficient analyses.

### Cluster analysis of qualitative traits

**FIGURE 5:**
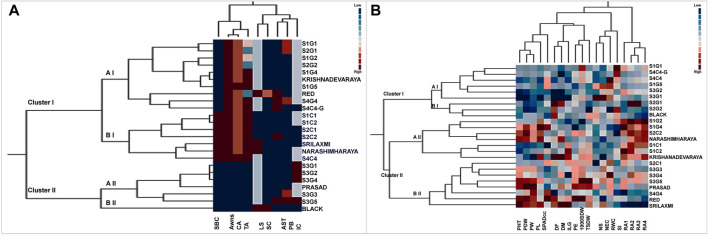
Two way cluster analysis for **(A)** qualitative and **(B)** quantitative traits in twenty foxtail millet landraces along with four released cultivars. Two way cluster analysis constructed double dendrograms, on the vertical direction, first dendrogram represent genotypes and horizontal direction second dendrogram representing traits. Colored bars indicated in the two-way cluster corresponding to the trait mean data.

Two way cluster analysis is a re-validation of PCA and the twenty-four foxtail millet genotypes were grouped into 2 major clusters I and II based on the Euclidean Ward approach (Figure 5A). Cluster I consisting of 17 genotypes further bifurcated into two groups-aI and bI. Sub-cluster aI included a mixture of landraces from various locations that clustered with the released cultivar, Krishnadevaraya. Surprisingly the colored genotypes from all locations, along with the released cultivars, Srilaxmi and Narashimharaya formed sub-cluster bI. Cluster II enclosed of seven genotypes subdivided into two main subclusters aII and bII. Sub-cluster aII contains S3G1, S3G2, S3G4, Prasad, S3G3 and S3G5. Sub-cluster bII contains only one genotype Black. In two-way clustering, nine qualitative traits also divided into two major clusters I and II. Clusters I enclosed color traits like SBC, BSL, CB and TB. Clusters II contain remaining traits which include the LS, SC, AST, PL and IC (Figure 5A).

### Cluster analysis of quantitative traits

Based on morpho physiological, yield and yield-related traits were grouped twenty-four genotypes into two major clusters I and II (Figure 5B). Cluster I consist of nine genotypes subdivided into two main subclusters aI and bI. Sub cluster aI included genotypes from various locations, whereas, sub cluster bI included S2G1, S2G2, and Black. Cluster II consists of fifteen genotypes sub divided into two main sub clusters aII and bII. Subcluster aII consists of seven genotypes namely S1G2, S1G4, S2C2, Narashimharaya, S1C1, S1C2, and Krishnadevaraya. Sub cluster bII consists of eight genotypes S2C1, S3G3, S3G4, S3G5, Prasad, S4G4, Red, and Srilaxmi. This cluster includes potential genotypes for crop improvement with traits like low days to flowering, days to maturity and high yielding genotypes. The second dendrogram of two-way clustering for quantitative traits displayed two major clusters I and II. Clusters I enclosed the four root angle traits whereas, other fifteen quantitative traits belongs to cluster II.

### Assessment of genetic diversity in the 24 foxtail millet genotypes

**FIGURE 6:**
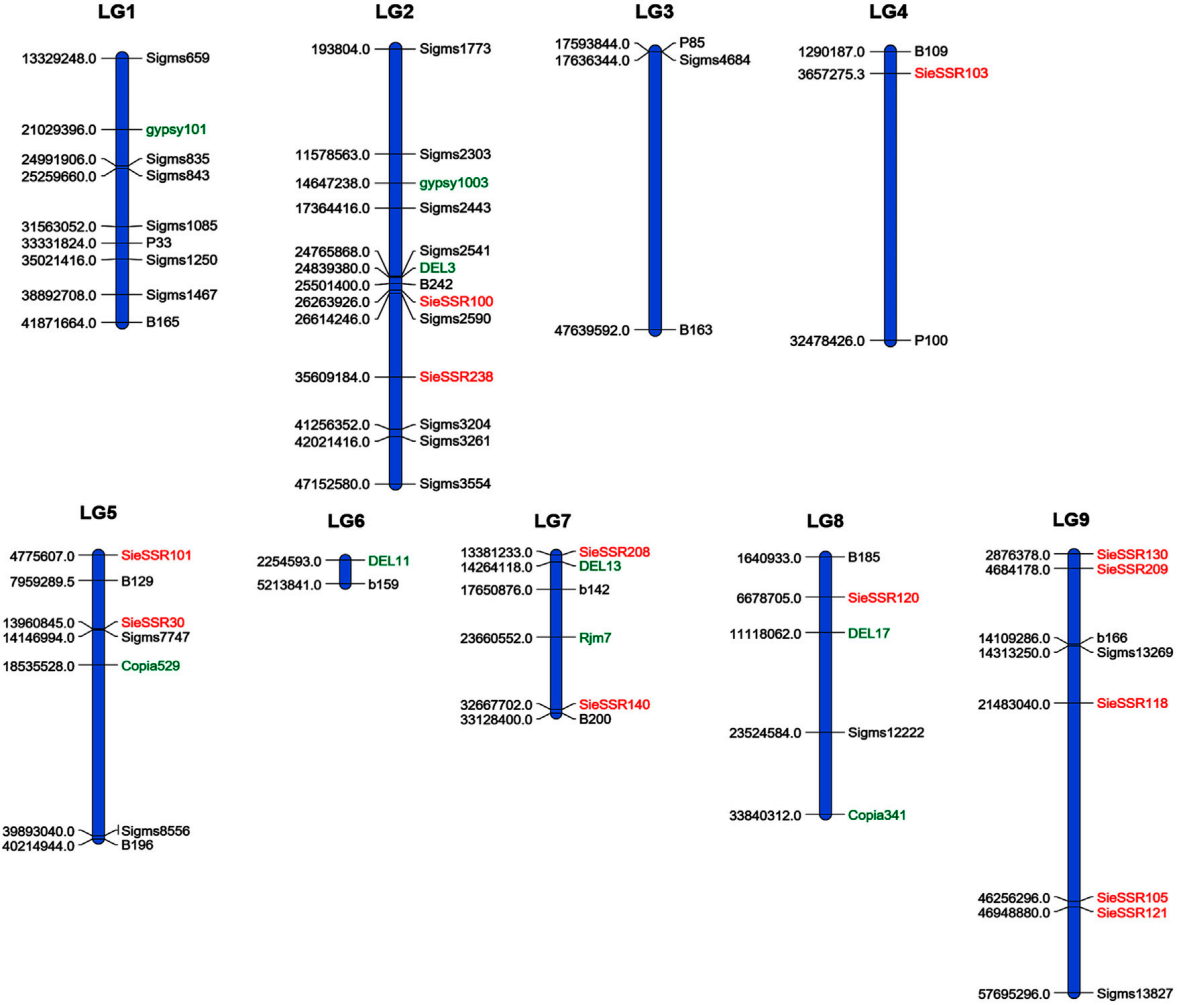
A total of 34 SSR, 21 EST SSR and 9 TE based markers dispersed across the 9 chromosomes to assess the genetic diversity in 24 foxtail millet genotypes. The markers labeled with black and red color were SSR and EST SSR markers and green color labeled markers were TE based markers.

A total of 34 SSR, 21 EST-SSR, and 9 TE based markers dispersed across the nine chromosomes were used to assess the genetic diversity in 24 foxtail millet genotypes except 3 rice SSR markers (Figure 6) and a total of 127 alleles were detected (Supplementary Figure S4). 127 different alleles were detected using these markers. Diversity analysis revealed the number of alleles per locus varied from 1 to 4, with a mean of 1.89 alleles per locus (Supplementary Table S9), whereas the effective number of alleles ranged from 1 to 3.25 with an average of 1.50. The various loci of the allele frequencies were differently distributed among the foxtail millet genotypes as shown in the table (Supplementary Table S9). Allelic frequencies showed wide variations, ranging from 0.02 to 1. Eleven rare alleles (8.66%) with a frequency <0.05 and 48 loci comprise 48 abundant alleles (37.79%) with frequency >0.50 remaining loci comprise 68 intermediate alleles (53.54%) with 0.05 < frequency <0.50 were detected. The PIC value for 67 markers varied from 0.0 to 0.47 with a mean of 0.22 (Table 4). The size of reproducible and scorable alleles ranged from 90 bp (Rm239) to 350 bp (B200) and detected a total of 127 alleles. The Shannon index ranged from 0 to 1.26 with an average of 0.40 (Table 4). The expected heterozygosity ranged from 0 to 0.70 with an average value of 0.25, whereas observed heterozygosity varied from 0 to 1 and with an average value of 0.23. Observed heterozygosity was 1 at two loci indicating high diversity in the foxtail millet genotypes (Table 4).

**TABLE 4:** Genetic characteristics of 20 foxtail millet landraces along with four released cultivars based on 67 markers used and their chromosome location, product size, number of polymorphic alleles, and PIC.

Marker ID	NA	NPA	PP	PIC	na*	ne*	I*	Obs_Hom	Obs_Het	Exp_Hom*	Exp_Het*	Nei**	Ave_Het
SieSSR30	2.00	2.00	100.00	0.42	2.00	1.95	0.68	0.17	0.83	0.50	0.50	0.49	0.49
SieSSR72	3.00	3.00	100.00	0.36	2.00	1.98	0.69	1.00	0.00	0.49	0.51	0.50	0.50
SieSSR96	3.00	3.00	100.00	0.39	3.00	2.44	0.97	0.29	0.71	0.39	0.61	0.59	0.59
SieSSR99	1.00	0.00	0.00	0.00	1.00	1.00	0.00	1.00	0.00	1.00	0.00	0.00	0.00
Sigms13269	4.00	4.00	100.00	0.39	4.00	3.26	1.27	0.05	0.95	0.29	0.71	0.69	0.69
Sigms2303	3.00	1.00	33.30	0.25	3.00	1.92	0.75	0.28	0.72	0.51	0.49	0.48	0.48
Sigms2541	1.00	0.00	0.00	0.00	1.00	1.00	0.00	1.00	0.00	1.00	0.00	0.00	0.00
Sigms3261	3.00	3.00	100.00	0.36	3.00	2.07	0.81	0.83	0.17	0.47	0.53	0.52	0.52
Sigms4684	2.00	1.00	50.00	0.24	2.00	1.55	0.54	0.63	0.38	0.64	0.36	0.35	0.35
Sigms659	1.00	0.00	0.00	0.00	1.00	1.00	0.00	1.00	0.00	1.00	0.00	0.00	0.00
Sigms843	2.00	2.00	100.00	0.12	2.00	1.13	0.23	0.96	0.04	0.88	0.12	0.12	0.12
Sigms8556	5.00	4.00	80.00	0.16	3.00	2.19	0.85	0.00	1.00	0.44	0.56	0.54	0.54
Sigms1085	3.00	3.00	100.00	0.21	3.00	1.43	0.56	1.00	0.00	0.69	0.31	0.30	0.30
SieSSR102	3.00	3.00	100.00	0.32	4.00	2.37	1.03	0.29	0.71	0.41	0.59	0.58	0.58
SieSSR244	1.00	1.00	100.00	0.15	1.00	1.00	0.00	1.00	0.00	1.00	0.00	0.00	0.00
SieSSR100	2.00	1.00	50.00	0.08	1.00	1.00	0.00	1.00	0.00	1.00	0.00	0.00	0.00
SieSSR101	1.00	0.00	0.00	0.00	1.00	1.00	0.00	1.00	0.00	1.00	0.00	0.00	0.00
SieSSR103	2.00	2.00	100.00	0.28	2.00	1.38	0.45	1.00	0.00	0.72	0.28	0.28	0.28
SieSSR105	1.00	0.00	0.00	0.00	1.00	1.00	0.00	1.00	0.00	1.00	0.00	0.00	0.00
SieSSR114	3.00	3.00	100.00	0.30	2.00	1.60	0.56	1.00	0.00	0.61	0.39	0.38	0.38
SieSSR115	2.00	2.00	100.00	0.35	2.00	1.52	0.52	1.00	0.00	0.65	0.35	0.34	0.34
SieSSR121	1.00	0.00	0.00	0.00	1.00	1.00	0.00	1.00	0.00	1.00	0.00	0.00	0.00
SieSSR140	4.00	4.00	100.00	0.36	4.00	2.87	1.20	0.65	0.35	0.33	0.67	0.65	0.65
SieSSR208	1.00	0.00	0.00	0.00	1.00	1.00	0.00	1.00	0.00	1.00	0.00	0.00	0.00
SieSSR209	3.00	1.00	33.30	0.17	2.00	2.00	0.69	0.00	1.00	0.48	0.52	0.50	0.50
SieSSR240	2.00	2.00	100.00	0.12	2.00	1.09	0.18	0.91	0.09	0.92	0.09	0.08	0.08
SieSSR118	1.00	0.00	0.00	0.00	1.00	1.00	0.00	1.00	0.00	1.00	0.00	0.00	0.00
SieSSR120	2.00	2.00	100.00	0.47	2.00	1.88	0.66	1.00	0.00	0.52	0.48	0.47	0.47
SieSSR130	3.00	3.00	100.00	0.36	3.00	2.70	1.04	0.08	0.92	0.36	0.64	0.63	0.63
SieSSR238	3.00	3.00	100.00	0.29	3.00	1.76	0.77	0.96	0.04	0.56	0.44	0.43	0.43
SiGMS8556	1.00	0.00	0.00	0.00	1.00	1.00	0.00	1.00	0.00	1.00	0.00	0.00	0.00
Sigms13827	3.00	3.00	100.00	0.26	3.00	1.65	0.68	1.00	0.00	0.60	0.40	0.39	0.39
Sigms3554	2.00	2.00	100.00	0.39	2.00	1.60	0.56	1.00	0.00	0.61	0.39	0.38	0.38
Sigms7747	3.00	3.00	100.00	0.33	3.00	1.85	0.81	0.95	0.05	0.53	0.47	0.46	0.46
Sigms835	1.00	0.00	0.00	0.00	1.00	1.00	0.00	1.00	0.00	1.00	0.00	0.00	0.00
Sigms1250	1.00	0.00	0.00	0.00	1.00	1.00	0.00	1.00	0.00	1.00	0.00	0.00	0.00
Sigms2590	2.00	2.00	100.00	0.28	2.00	1.38	0.45	1.00	0.00	0.72	0.28	0.28	0.28
Copia529	1.00	0.00	0.00	0.00	1.00	1.00	0.00	1.00	0.00	1.00	0.00	0.00	0.00
Copia341	1.00	0.00	0.00	0.00	1.00	1.00	0.00	1.00	0.00	1.00	0.00	0.00	0.00
DEL11	2.00	2.00	100.00	0.32	2.00	1.71	0.61	0.41	0.59	0.57	0.43	0.42	0.42
DEL13	1.00	1.00	100.00	0.41	1.00	1.00	0.00	1.00	0.00	1.00	0.00	0.00	0.00
DEL17	1.00	1.00	100.00	0.28	1.00	1.00	0.00	1.00	0.00	1.00	0.00	0.00	0.00
DEL3	3.00	3.00	100.00	0.34	3.00	2.39	0.98	0.43	0.57	0.37	0.63	0.58	0.58
gypsy101	1.00	0.00	0.00	0.00	1.00	1.00	0.00	1.00	0.00	1.00	0.00	0.00	0.00
gypsy1003	2.00	2.00	100.00	0.25	2.00	1.87	0.66	0.26	0.74	0.52	0.48	0.47	0.47
Rjm7	5.00	3.00	60.00	0.10	2.00	2.00	0.69	0.00	1.00	0.47	0.53	0.50	0.50
B163	1.00	1.00	100.00	0.22	1.00	1.00	0.00	1.00	0.00	1.00	0.00	0.00	0.00
B165	2.00	2.00	100.00	0.30	1.00	1.00	0.00	1.00	0.00	1.00	0.00	0.00	0.00
B109	2.00	2.00	100.00	0.08	2.00	1.14	0.24	0.87	0.13	0.88	0.12	0.12	0.12
B129	1.00	1.00	100.00	0.28	1.00	1.00	0.00	1.00	0.00	1.00	0.00	0.00	0.00
b142	3.00	3.00	100.00	0.42	3.00	2.45	0.99	0.16	0.84	0.39	0.61	0.59	0.59
b159	3.00	3.00	100.00	0.33	2.00	1.10	0.18	0.91	0.09	0.91	0.09	0.09	0.09
b166	1.00	1.00	100.00	0.47	2.00	1.92	0.67	0.88	0.13	0.51	0.49	0.48	0.48
B185	3.00	3.00	100.00	0.22	3.00	2.21	0.86	0.05	0.95	0.44	0.56	0.55	0.55
b196	2.00	2.00	100.00	0.38	2.00	1.52	0.52	1.00	0.00	0.65	0.35	0.34	0.34
B269	2.00	2.00	100.00	0.08	2.00	2.00	0.69	0.04	0.96	0.49	0.51	0.50	0.50
P100	4.00	4.00	100.00	0.30	3.00	2.06	0.85	0.50	0.50	0.47	0.53	0.51	0.51
p80	2.00	2.00	100.00	0.24	2.00	1.10	0.19	1.00	0.00	0.91	0.09	0.09	0.09
P6	2.00	2.00	100.00	0.08	2.00	2.00	0.69	0.00	1.00	0.49	0.51	0.50	0.50
p85	1.00	1.00	100.00	0.08	1.00	1.00	0.00	1.00	0.00	1.00	0.00	0.00	0.00
B234	3.00	3.00	100.00	0.23	3.00	1.52	0.62	1.00	0.00	0.65	0.35	0.34	0.34
B200	1.00	1.00	100.00	0.41	1.00	1.00	0.00	1.00	0.00	1.00	0.00	0.00	0.00
B242	1.00	1.00	100.00	0.38	1.00	1.00	0.00	1.00	0.00	1.00	0.00	0.00	0.00
p33	1.00	1.00	100.00	0.49	1.00	1.00	0.00	1.00	0.00	1.00	0.00	0.00	0.00
Rm257	1.00	1.00	100.00	0.27	1.00	1.00	0.00	1.00	0.00	1.00	0.00	0.00	0.00
Rm263	1.00	1.00	100.00	0.15	1.00	1.00	0.00	1.00	0.00	1.00	0.00	0.00	0.00
Rm239	2.00	2.00	100.00	0.37	2.00	1.54	0.53	0.55	0.45	0.64	0.36	0.35	0.35

NA, number of amplicans; NPA, number of polymorphic amplicans; PP, percentage of polymorphism; PIC, polymorphic information content; na*, observed number of alleles; ne*, effective number of alleles; I*, shannon’s information index; He, expected heterozygosity; Ho, observed heterozygosity; Obs_Hom, observed homozygosity; Obs_Het, observed heterozygosity; Exp_Hom, expected homozygosity; Exp_Het, expected heterozygosity; Nei**, gene flow; Ave_Het, average heterozygosity.

### Molecular cluster analysis

**FIGURE 7:**
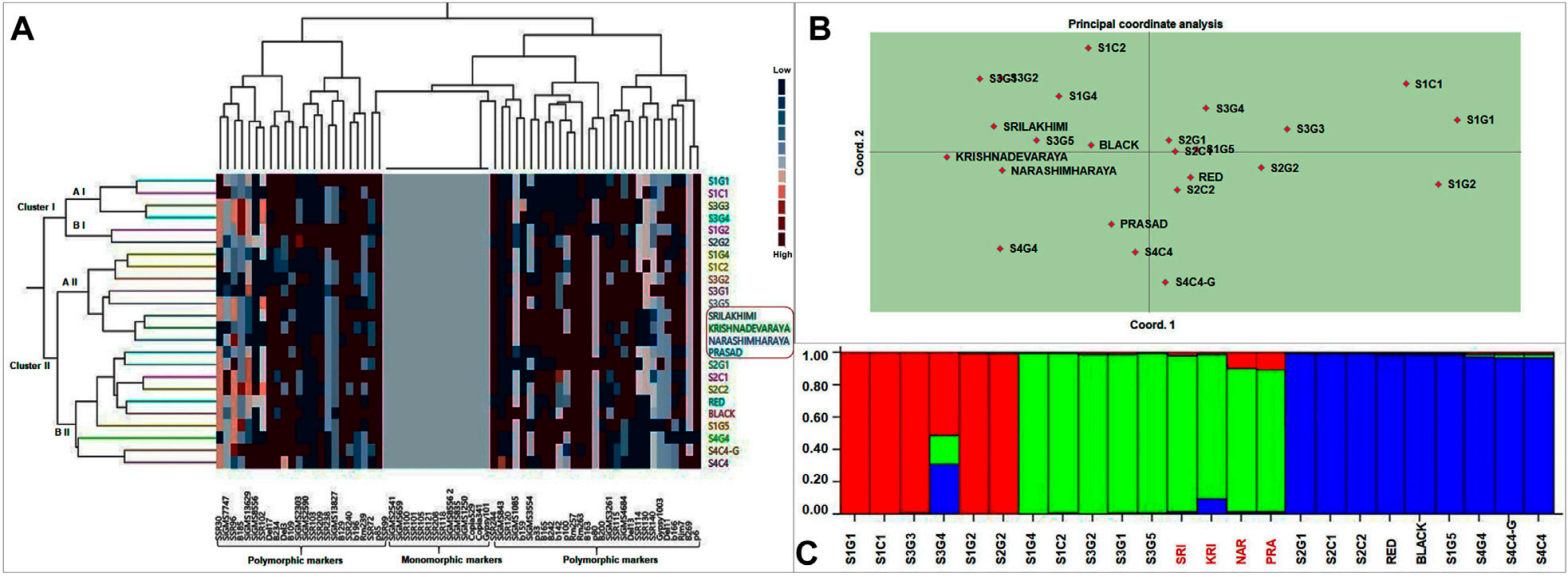
**(A)** Phylogenetic relationships between landraces and released cultivars of foxtail millet based on Euclidean Ward’s method of similarity coefficients computed from data matrix with polymorphic DNA bands generated from 67 molecular markers. In two way cluster analysis, first dendrogram represent 24 foxtail millet genotypes and second dendrogram representing molecular markers (34 SSR, 21 EST SSR, 9 TE based and 3 rice SSR markers). Colored bars indicated in the two-way cluster corresponding to the marker amplification data. **(B)** Principal Coordinate analysis and **(C)** population structure analysis of the twenty foxtail millet landraces along with four released cultivars based on 127 alleles generated from molecular markers.

A dendrogram was constructed by cluster analysis (Euclidean Ward’s method) to reveal the genetic relationships between landraces and cultivars, based on the 127 genotyping amplicons generated in this study. These 24 foxtail millet genotypes were separable into two major clusters, cluster I (6 genotypes) and cluster II (18 genotypes), with the latter including two prominent subclusters, a II (8 genotypes) and b II (10 genotypes) (Figure 7A). The dendrogram reveals that the genotype clustering was partly driven by geographical location as well as genotypic similarity. Nearly all genotypes from location 1 and 3 separated into cluster I and a II, whereas all genotypes from locations 2 and 4 formed subcluster b II. Interestingly, nearly all high yielding landraces (except S1C1) and released cultivars accumulated in cluster II (Figure 7A). This is consistent with the observation that cluster I contained mono and polymorphic markers whereas cluster II contained only polymorphic markers, suggesting a correlation of the latter with higher yield and performance.

### Principal coordinate analysis (PCoA)

PCoA was also performed to find genetic relationships among landraces and commercial varieties. The location of individuals was defined by PC 1 and PC 2, which combined to explain less than 10% of the variation; PC 1 and PC 2 explained 5.3% and 4.5% variation, respectively (Figure 7B). This implies that several more principal components are required to substantially explain the variance. As a result, the clustering of the genotypes is significantly less clear and this is likely due to pronounced inter-genotype differences (for example in the top right of the biplot) causing them to space out in the biplot. The lack of clear clustering with PCoA is not surprising as the intended purpose of PCoA is dimensionality reduction and not clustering, which is simply a secondary consequence of the former. Nevertheless, as with the molecular cluster analysis, most genotypes found within a region tended to congregate, resulting in a grouping pattern that resembled the cluster analysis. Cluster I genotypes from clustering analysis spread out on the top right of the PCoA biplot, subcluster a II clustered tightly to the top left and subcluster b II components aggregated towards the lower middle of the plot, resembling the overall topology of the clustering dendrogram. PCoA also clarifies that the best performing landrace (high yield, early flowering/maturity), S1C1 is genetically most distinct, while the other high yielding landraces (S3G5, Red and Black) clustered closely with the released cultivars.

### Population structure analysis

STRUCTURE 2.3.4 employing Bayesian clustering was used to decipher the population structure genetic relationships among foxtail millet genotypes (Figure 7C). Populations were studied for the number of pure and admix individuals. Simulations were accompanied with K value of 1–10 with 10 repetitions based on admixture model in the 24 genotypes which showed best structure model at K = 3. Thus, at K = 3, the model-based structure analysis classified the 24 foxtail millet genotypes into three sub-populations, which match the three clusters/sub-clusters (I, a II, b II) identified by cluster analysis (Supplementary Figure S5). Cluster I (primarily red in Figure 7C) included six landraces with three pure genotypes and three admixes from locations 1 and 3 and the same genotypes were found in cluster I of Ward’s clustering. Cluster II included five landraces and four released cultivars with three pure genotypes and six admixes and included nearly the same genotypes as cluster a II in Ward’s clustering. Cluster III had nine landraces with six pure and three admixes and the composition was nearly identical to cluster b II in Ward’s clustering. Compared to all twenty foxtail millet landraces along with four released varieties S1G1, S1G4, S1G5, S1C1, S2G1, S2C1, S2C2, S3G3, S3G5, Red, and Black are pure lines and S3G4, Krishnadevaraya, Narashimharaya and Prasad are most admixes. Notably, the best performing varieties identified (S1C1, S3G5, Red and Black) are pure genotypes.

## Discussion

Millets like foxtail millet are minor cereals widely farmed by resource-poor farmers in semi-arid regions of Asia and Africa. Even though foxtail millet is more nutritious, climate-resilient, more compatible with infertile soil than wheat and rice. Its production and research have been neglected, perhaps partly due to its poor yield (Sagar et al., 2020). Therefore, improving millet yield and resilience to stress is an urgent issue (Zhao et al., 2020). Foxtail millet productivity and production in the semi-arid region of Rayalaseema, India, faces immense challenges of drought and heat stress. Small holder and resource-poor farmers in Rayalaseema region struggle to acquire pure seed of landraces from a semiformal seed distribution system. To our knowledge, foxtail millet landraces and farmer varieties in India are poorly characterized and underutilized. In this study, we report the systematic collection, pure line development and characterization of foxtail millet landraces in the semi-arid regions of Rayalaseema, India (Figure 1).

### Phenotypic diversity of the landraces

An understanding of morphogenetic variation in agronomic traits of a crop can assist in the identification of superior lines with desirable traits of high yield, abiotic stress tolerance and biotic resistance (Santalla et al., 2001; Babu et al., 2017). Landraces are valuable assets for the development of high yielding and stress tolerant cereal crops and for the identification of novel alleles for these traits (Marone et al., 2021). To this end, we tapped into indigenous landraces of foxtail millet in South India and found a diverse genetic base, while purifying four superior landraces with high yielding and early flowering/maturing for crop improvement (Supplementary Tables S4, S6). In foxtail millet landraces, phenotypic analysis on morphological characteristics demonstrated high diversity (Reddy et al., 2006). In this study, analysis of 28 morpho-physiological yield and yield-related traits revealed striking phenotypic variation among the foxtail millet landraces (Figure 2; Supplementary Figures S2, S3; Table 2; Supplementary Tables S3, S4). The traits D50%F, DM, SI and TSDW displayed high estimates of heritability coupled with high genetic advance as percent of mean, suggesting additive gene effect and a higher genetic than environmental influence on these traits (Table 3). Both correlation and path coefficient analysis complemented these results as D50%F and DM strongly and directly impacted grain yield (Figure 3). RA 3, PDW, PE and PWT showed the highest variation and also correlate with and directly impact seed yield and therefore, may be criteria to select high yielding foxtail millet accessions. This formed the basis for identifying the early flowering and maturing, high yielding landraces- S1C1, S3G5, Red and Black in this study (Supplementary Table S6). These findings were in agreement with previous reports (Ashfaq et al., 2003; Prasad et al., 2006a; Prasad et al., 2006c). Moreover, these landraces provide a gene pool to identify novel stress-related alleles and could be used to develop biparental recombinant and advanced mapping populations for identification of genes for target traits (Kölliker et al., 2003; Rasheed et al., 2014; Upadhyaya and Vetriventhan, 2018).

For qualitative traits, first two components of PCA, accounted for 61.86% of the variation which suggests as a good correlation between these characters (Figure 4). Remarkably, panicle and bristles characteristics alone along with stem base color explained nearly 40% of the morphological variation, thus being the most significant contributor. Remarkably, Euclidean Ward clustering based on qualitative traits revealed segregation of all colored genotypes including the best performing landrace S1C1 and the pigmented released high yielding cultivars Srilakshmi and Narasimharaya into one cluster (bI), revealing the discriminatory power of color traits (Figure 5A). Plant coloration could, in some cases, be linked with drought tolerance and it would be exciting to explore this avenue to identify high yielding drought tolerant genotypes. PCA for the quantitative traits including root angles, plant height, dry weight and panicle exertion and panicle weight explained 40% of the variation (first two components of PCA), suggesting that the variation was a cumulative effect of a number of traits (Figure 4). Earliness to flowering and panicle characteristics which could impact fertility and yield were the traits with most discriminatory power, explaining the variability among the landraces and could be utilized for crop improvement.

### Genetic diversity of the landraces

Natural genetic diversity provides the raw material for plant breeding and understanding the diversity in available germplasm is a prerequisite to utilizing them for breeding and crop improvement (Upadhyaya et al., 2008). Higher genetic diversity in landraces can be utilized for the development of genetic markers, generate advanced segregating populations, and identification of novel genes for desirable traits and for varietal development (Chaudhary and Singh, 1982; Hirano et al., 2011; Lin et al., 2012; Wang et al., 2012; Fu, 2015). Detailed characterization of foxtail millet landraces is, thus, indispensible for identification genetically diverse genotypes for novel genes.

In this study, 67 SSR, EST-SSR and TE-based markers was employed for assessing the genetic relatedness, diversity and population structure of the landraces. SSR markers, were preferred as they are known to be more informative than other markers (Vieira et al., 2016). A total of 127 alleles were amplified (Supplementary Figure S4) and the average number of alleles per locus among the landraces in this study was 1.89 (Supplementary Table S9), which is comparable to or lower than other studies involving foxtail millet, where the alleles per locus ranged from 2.1 to 16.69 (Jia et al., 2009; Lin et al., 2012; Gupta et al., 2013; Pandey et al., 2013; Gupta et al., 2014; Chander et al., 2017). PIC is a better indicator of genetic diversity than alleles per locus as it also takes into account the frequency of the alleles. Along with the number of alleles per locus, the average PIC of 0.23 in this study (Table 4) indicates nearly moderate genetic diversity among the landraces (Botstein et al., 1980). The average PIC value in the present study was comparable to earlier reports (Gupta et al., 2012; Kim et al., 2012; Lin et al., 2012; Wu et al., 2014). Some of the variation observed in this study was in EST-SSRs, which may be linked to expressed, functional genes, making functional analysis of desired traits possible. Like PIC, expected heterozygosity (He) also measures of genetic diversity among genotypes. In our study, the expected heterozygosity ranged from 0 to 0.70 with an average value of 0.25, while the observed heterozygosity varied 0 to 1 with an average value of 0.23 (Table 4). Our results were in accordance with previous reports (Zhao et al., 2012; Gupta et al., 2014). The mean observed heterozygosity and Shannon’s information index (0.4) in our study (Table 4) also point to a medium level of genetic variation in the landraces. The lower Ho compared to He is not surprising considering the generally self-pollinated and inbreeding nature of foxtail millet. To start with, foxtail millet suffers from a small genetic base, aggravated by inbreeding and the self-pollinated nature of the crop (Lin et al., 2012). The moderation in genetic diversity, therefore, may be due to a relatively narrow genetic base of the landraces as the collections were made from relatively proximal locations (one to two latitude and longitude differences) in South India or due to common ancestry of the landraces. Gene flow resulting from the exchange of planting material among farmers could also reduce genetic diversity. Thus studies such as this, utilizing molecular markers to explore genetic diversity, not only serve to broaden the genetic base of foxtail millet for crop improvement, but also provide raw material for outbreeding. Allelic frequencies showed wide variations, ranging from 0.02 to 1 (Supplementary Table S9). A total of eleven rare alleles, 68 intermediate alleles and 48 abundant alleles were detected. Presence of rare alleles in landraces could be useful in identifying trait associations through allele mining and also used as a diagnostic marker for specific varietal identification. These alleles may also contain adaptive traits in specific genotypes (Upadhyaya et al., 2011). Using 223 core collection of foxtail millet accessions 11 unique alleles identified (Chander et al., 2017). Allelic variability among the 67 molecular markers was high enough to categorize foxtail millet genotypes, and to catalog the genetic variability observed for future use. In previous studies, sufficient information on genetic markers for DNA polymorphisms found a higher level of molecular diversity (Jia et al., 2009; Yadav et al., 2014). We also tested rice molecular markers in the landraces and found that all the tested primers worked indicating that the markers are transferable across species and conserved traits may be examined in foxtail millets.

Molecular markers can also be employed to assess the phylogenetic relationship between genotypes. Ward’s clustering analysis, principal coordinate analysis and population structure analyses based on the molecular markers partitioned the landraces into two to three subpopulations or genetic groups, partly according to the genotypes and partly corresponding with their geographic location (Figure 7). In addition to cluster analysis, PCoA revealed a grouping pattern that resembled the genotypic cluster analysis (Figure 7B). Finally, structure analysis also revealed twenty landraces along with released cultivars separated into three sub populations (12 pure and 12 admixtures) (Figure 7B). The composition of the three clusters in Structure is nearly identical to those in Cluster I and the two subclusters in Cluster II, indicating a concordance between the analyses. The limited geographical separation of the genotypes is consistent with a high proportion of admixtures. This may be due to the frequent exchange of foxtail millet landraces from one region to other region. This result is agreement with previous studies (Vetriventhan et al., 2014; Ali et al., 2016). Some earlier reports did find that foxtail millet landraces were separated based on geographical origins (Hirano et al., 2011).

## Conclusion

The present work highlights 20 foxtail millet landraces development and evaluated along with four released cultivars. Of the 9 qualitative and 19 quantitative traits, days to flowering, days to maturity and panicle characteristics contribute significantly towards the genetic divergence and these traits can be utilized in breeding programs for crop improvement. Polymorphic marker, identified in the study is useful in marker assisted selection and rare alleles can be used as a marker trait association and diagnostic marker for specific varietal identification. A moderately high phenotypic and genotypic diversity was observed in landraces, which is an indication of a diverse gene pool. Among the 20 developed landraces short-duration high yielding superior lines namely S3G5, Red, Black and S1C1 were identified. These superior lines have been selected for multi-location trials for its high yield and high stability performance. These stable landraces may be recommended for varietal release. This study also highlights the value of landraces and demonstrates that a wider geographical coverage and a more diverse panel of markers could unearth a goldmine of genetic resources for millet crop improvement for low-input sustainable agriculture.

## Data Availability

The original contributions presented in the study are included in the article/Supplementary Material, further inquiries can be directed to the corresponding authors.

## Supplementary material

SUPPLEMENTARY FIGURE S1 Collection sites of foxtail millet germplasm used in this study in Rayalaseema region in South India.

SUPPLEMENTARY FIGURE S2 Morphological traits variability observed in twenty landraces of foxtail millet along with four released cultivars under study for panicle variation and root angles.

SUPPLEMENTARY FIGURE S3 Variability of stomatal number observed in twenty landraces of foxtail millet along with four released cultivars under study.

SUPPLEMENTARY FIGURE S4 Molecular marker genotyping of twenty foxtail millet landraces along with four released cultivars under study.

SUPPLEMENTARY FIGURE S5 The detection of the true number of clusters (the most likely value of K) inferred by the STRUCTURE software and set ΔK = mean (|L″(K)|)/sd (L(K)) as a function of K. ΔK achieves its highest peak when K = 3 generated by the STRUCTURE HARVESTER.
