# Supplementary figures and images for: Molecular genetics and phenotypic assessment of foxtail millet (Setaria italica (L.) P. Beauv.) landraces revealed remarkable variability of morpho-physiological, yield, and yield‐related traits
Source: Front Genet. 2023 Jan 25;14:1052575. doi: 10.3389/fgene.2023.1052575 (PMC9905688; doi:10.3389/fgene.2023.1052575)

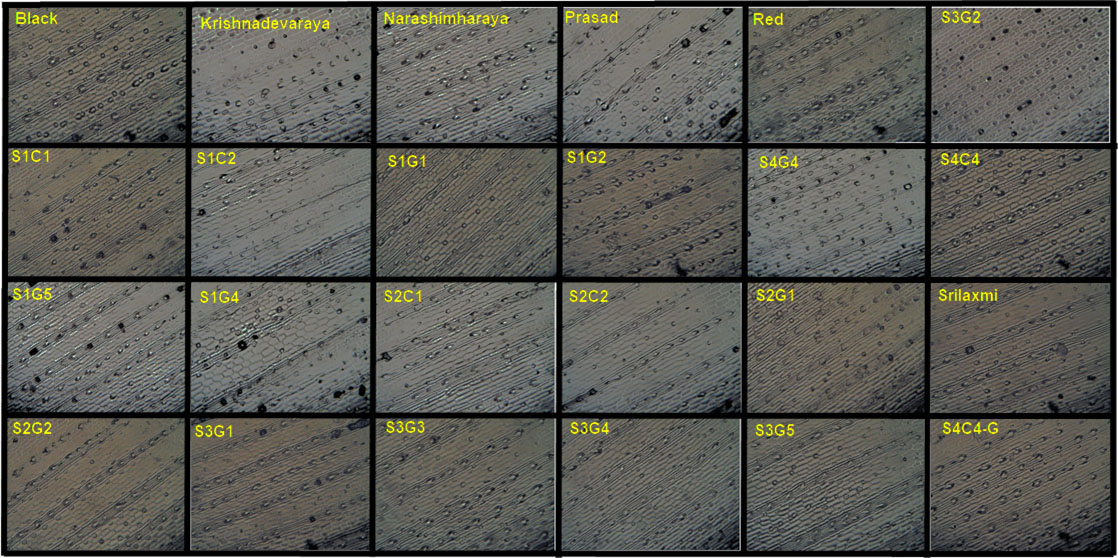

Supplement: Supplementary file 1 [file Image3.JPEG]

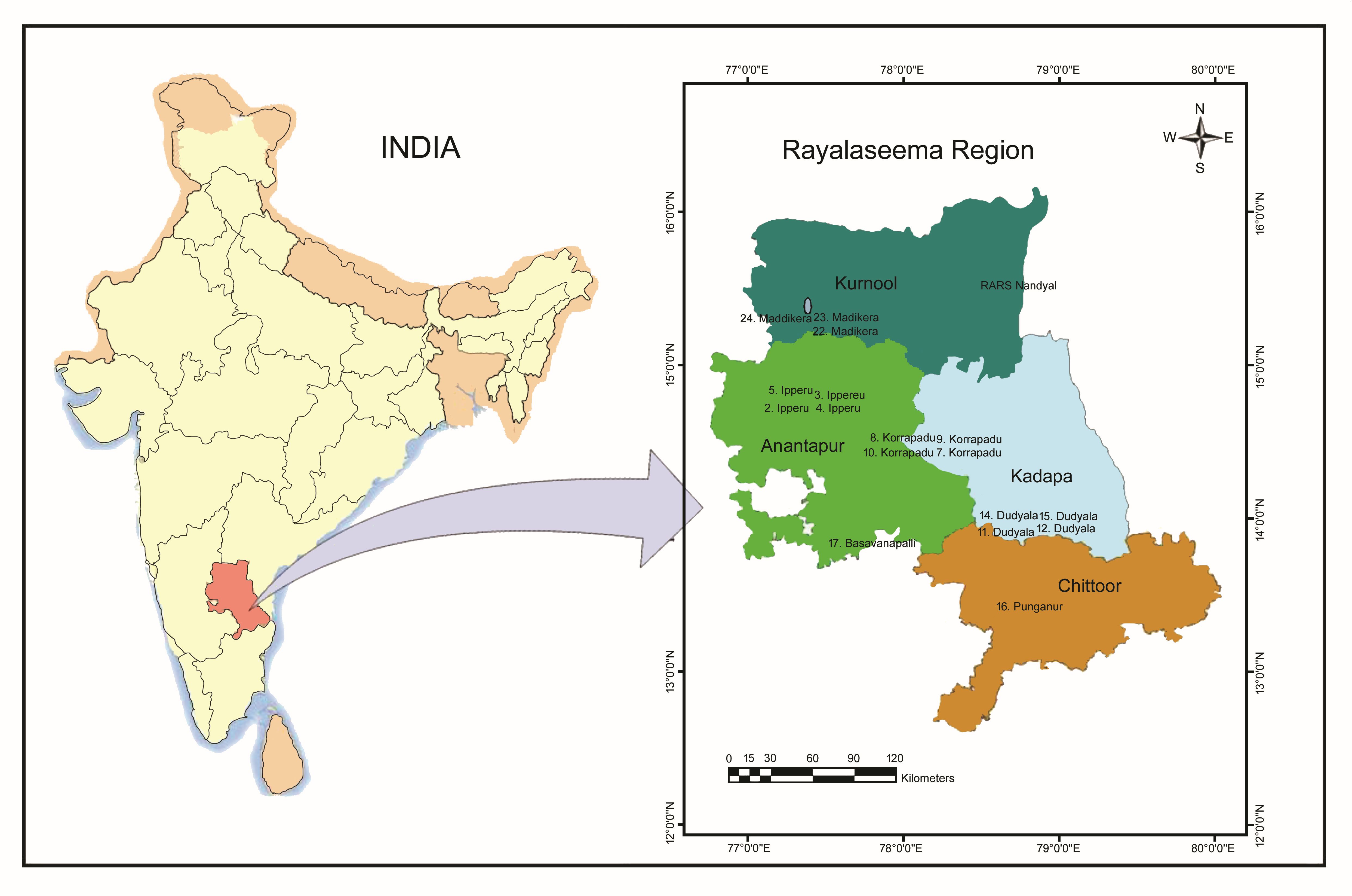

Supplement: Supplementary file 2 [file Image1.JPEG]

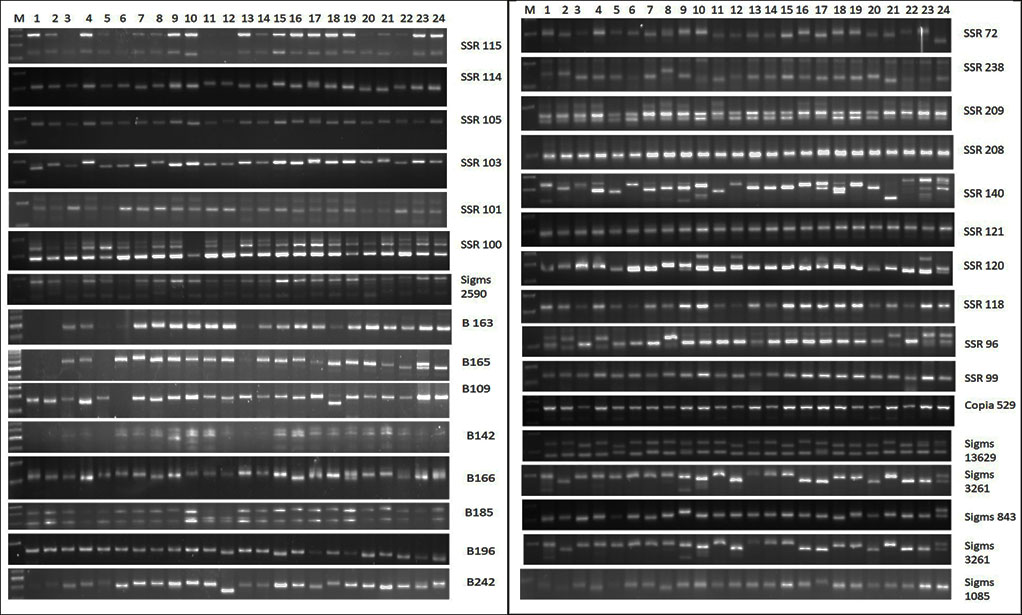

Supplement: Supplementary file 3 [file Image4.JPEG]

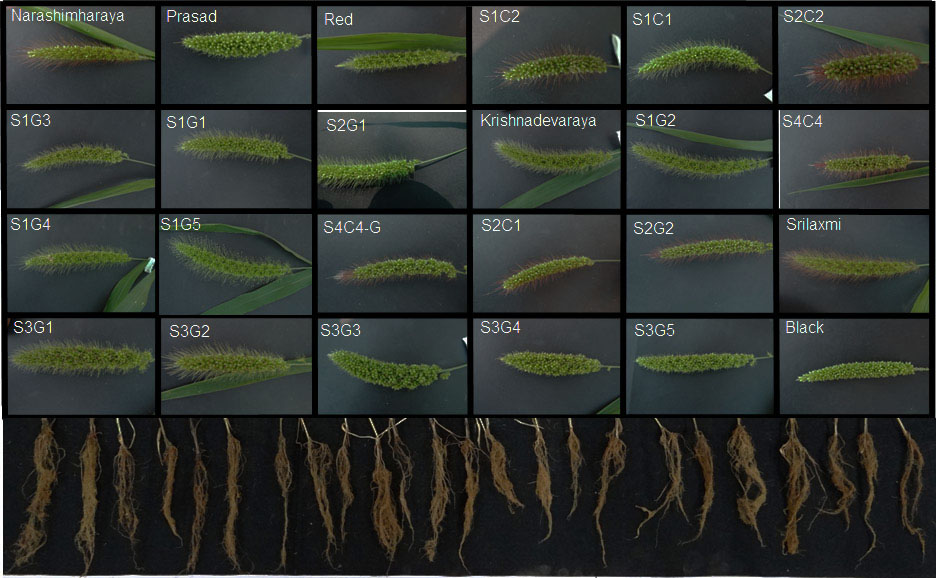

Supplement: Supplementary file 4 [file Image2.JPEG]

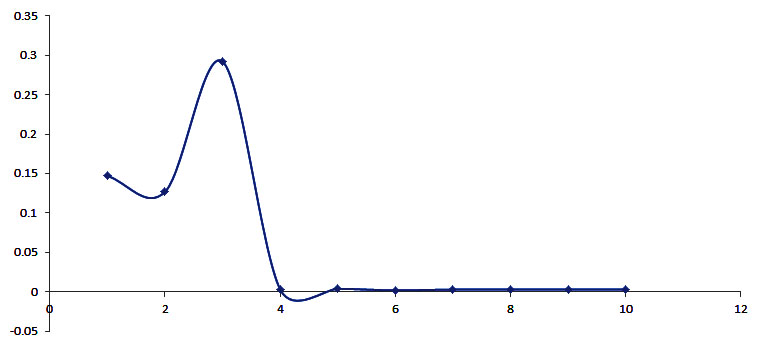

Supplement: Supplementary file 5 [file Image5.JPEG]
